# Clinical and Ethical Challenges in Undertaking LIMPRINT in Vulnerable Populations

**DOI:** 10.1089/lrb.2018.0083

**Published:** 2019-04-22

**Authors:** Christine J. Moffatt, Martina Sykorova, Aimee Aubeeluck, Peter John Franks, Sarah Pankhurst, Rachel Bussey, Siobhan Whiston, Susie Murray, Gregoire Mercier, Isabelle Quéré, Susan Gordon

**Affiliations:** ^1^ School of Social Sciences, Nottingham Trent University, Nottingham, United Kingdom.; ^2^ Montpellier Medecine Vasculaire, EA2992, Universite Montpellier I, Montpellier, France.; ^3^ Copenhagen Wound Healing and Lymphoedema Centre, Bisperberg University Hospital, Copenhagen, Denmark.; ^4^ Nottingham University Business School, University of Nottingham Jubilee Campus, Nottingham, United Kingdom.; ^5^ School of Health Sciences, Queens Medical Centre, University of Nottingham, Nottingham, United Kingdom.; ^6^ Centre for Research & Implementation of Clinical Practice, London, United Kingdom.; ^7^ Nottingham CityCare Partnership Cic and Tissue Viability Services, Nottingham, United Kingdom.; ^8^ Nottinghamshire Healthcare NHS Foundation Trust, Duncan MacMillan House, Nottingham, United Kingdom.; ^9^ College of Nursing and Health Sciences, Flinders University and ACH Group, Adelaide, South Australia.

**Keywords:** prison population, residential care facility, residential home, vulnerable populations, prevalence, lymphedema, lymphoedema

## Abstract

***Background and Study Objective:*** To estimate the prevalence of chronic edema (CO) and wounds within two vulnerable populations, a male high security prison in the East Midlands (United Kingdom) and residential and nursing homes in the United Kingdom and Australia.

***Methods and Results:*** Methods for screening for CO and wounds were adapted from the main LIMPRINT methodology.

***Prison Population:*** In total, 195 inmates were recruited with 22 (11%) having CO. While the majority were white Caucasian (156/83.4%) a further 20 (10.7%) were dark skinned with 11 (5.95%) from other minority populations. Comorbidities included 123 (63%) smokers, 22 (11%) alcohol dependant, 60 (31%) with mental health problems, and 35 (18%) a history of self-harm. Only three had a current wound with 30 (16%) having had a traumatic stab wound.

***Residential and Nursing Homes (United Kingdom and Australia):*** In the United Kingdom, the total population available for inclusion was 189 with only 137 (73%) recruited. Seventy-two of the 137 (52%) suffered from CO and a further 16 (23%) had a history of cellulitis. Results from the Australian residential care facilities have been published in full. In summary, of the 37 participants 20 (54%) experienced CO with 25 (68%) having comorbidities and 11 (30%) having a concurrent wound.

***Conclusion:*** Obtaining an accurate picture of the prevalence and impact of CO in vulnerable populations is extremely challenging due to issues of access and consent. Lack of reliable data for these populations will contribute to poor service provision.

## Background

This article addresses the complex ethical challenges faced during LIMPRINT, an international epidemiology study to determine the size and impact of chronic edema (CO) (swelling present >3 months) within complex health and social care settings and the implications this has for adoption of a public health approach to care provision. Studies were attempted in a range of settings in the United Kingdom, including nursing and residential care homes, social care services at home, a large male prison, and an acute mental health institution. Complex issues, including capacity and inability to provide informed consent, limit a true appreciation of the size and impact of the problem in vulnerable patient groups despite the likelihood of them having a high prevalence due to known comorbidities and risk factors. Issues of professional knowledge and gatekeeping of patients were difficult and led to recruitment difficulties. A similar ethical dilemma occurred in Australia when attempting to access vulnerable people living in aged care facilities. Issues of professional knowledge and gatekeeping of patients led to recruitment difficulties.

## Introduction

### LIMPRINT background

#### Limprint—(Lymphoedema IMpact and PRevalence–INTernational: International Lymphoedema Framework)

CO is a major clinical problem worldwide, which has many important secondary consequences. The term “chronic edema” is now commonly used in place of “lymphedema” as this encompasses all forms of edema, which persist for 3 months or longer, irrespective of the etiology and corresponding comorbidities and risk factors.^1^ Although CO has potentially life-threatening consequences, the prevalence and impact of the problem remain poorly understood.^2^ LIMPRINT was an international epidemiology study that was designed to define the prevalence and impact of CO in health services in different countries and health care systems. The development and validation of the methods and main results are reported separately.^3,4^

#### Background to the ethical challenges in assessing vulnerable populations

The LIMPRINT methodology was designed to be able to capture data about people with CO in any care setting using a core tool and simple screening procedure to confirm the presence or absence of CO. However, despite this, there are many challenges faced in accessing vulnerable patient groups who are unable to provide informed consent in settings such as prisons, nursing homes, aged care facilities, and mental health facilities. When seeking to undertake a public health approach to understanding the needs in the population a failure to access these groups could lead to a serious underestimate of the true size and impact of the problem. The case studies presented in this article illustrate these complex issues and the ethical and practical challenges researchers faced. While the main core research from LIMPRINT was undertaken in a range of acute hospitals, specialist lymphedema services, general and specialist outpatient services, and community nursing services, no information was available concerning the prevalence in nursing and residential homes. However, due to the known risk factors of age and increased comorbidities it was hypothesized that both CO and wounds of varying types may occur more frequently as well. As well prevalence of CO and wounds within prisons was largely unknown, with an Irish study reporting increased rates of mental illness and wounds due to self-harm and leg ulceration from mainline drug use causing venous ulceration in a prison population.^5^

The partners involved in this work wished to undertake the study as part of a service development program. Studies were planned to complete LIMPRINT in mental health and social care settings in the East Midlands, United Kingdom. LIMPRINT methodology has also been incorporated into a study of patients attending a bariatric service and a population with Multiple Sclerosis in the United Kingdom. Both these studies will be published separately.

The following aims were defined for these specialist patient populations:

- To estimate the prevalence, impact, and risk factors associated with CO and to determine the proportion with concurrent wounds in the following settings:
- Male, high security prison in the East Midlands (United Kingdom)
- Residential and nursing homes in the United Kingdom and Australia
- Residential Mental Health Services in the East Midlands (United Kingdom)
- Social care services in the East Midlands (United Kingdom)

Each study will be presented as an individual case study and the results and challenges discussed.

### Data analysis

Anonymized data from each study site were entered onto an Excel spreadsheet and exported into Stata 11 where statistical analyses were undertaken. The analysis included the generation of crude prevalence rates according to the signs and symptoms of CO. Logistic regression analysis was undertaken to examine the relationship between the presence of CO and demographic details (age and gender) where appropriate, together with comorbidities, including obesity and poor mobility. Finally, an analysis was undertaken to identify independent factors that were associated with the presence of CO in each cohort.

## Case Study 1: Prison Service Prevalence

### Background

Little attention has been placed on the problem of wounds and CO within a prison setting. It can be postulated that they represent a younger population with a different risk factor profile than in other health care settings. There is a likelihood of mental health problems leading to self-harm and drug and alcohol dependency. Mainline intravenous drug use (injection of drugs such as heroin) is associated with the development of severe venous disease leading to venous ulceration. This study formed part of a wider health service development program to improve the assessment and management of the general health care for prisoners.

## Methods

### Setting

The study was undertaken in a category B (high risk) male prison in the East Midlands (United Kingdom) with an operational capacity to house 1060 inmates at any one time. It is a short stay prison facility with four main residential wings.

### Identification of patients

**FIG. 1.**
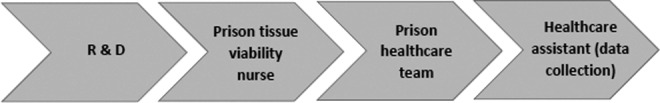
The gatekeeping process of accessing patients, prison study.

The study was considered a service evaluation and therefore did not require a full ethics approval but received research and governance approval to undertake the project. However, prison inmates were required to provide informed written consent due to the vulnerability of their status and the research-governance requirements for the prison population. Accessing patients in this study was complex (Fig. 1) and required a number of gatekeepers to facilitate the process.

### Preparation and training of staff and study sample

Due to the high security risk associated with the types of prisoners in this study, no research staff were allowed into the prison facilities. This required that all training and monitoring were performed by the prison health service. The study was overseen by the Prison tissue viability nurse specialist, and a dedicated health care assistant was allocated to the project and trained in the screening methods (stemmer sign, pitting test, and assessment of wounds). The screening and assessment methods have been previously published.^3^ The study ran for 2 months, and issues of prison capacity and access to a treatment room led to recruitment from only two wings.

### Informed consent

The recruited health care assistant obtained written consent from each participant who was provided with information the night before the assessment took place. All consent forms were stored within the prison health care facilities. Each participant was given a small bag of sweets as a thank you for participating.

## Results

During the study period 195 inmates were recruited with a further 54 declining to participate for a range of reasons. The rate of swelling at any site on the body was low (22/11%) with only 5 participants having swelling/pitting edema and 17 having a positive stemmer sign suggestive of chronic skin changes. Two other inmates were reported to have a history of cellulitis. While the majority were white Caucasian (156/83.4%) a further 20 (10.7%) were dark skinned with 11 (5.95%) from other minority populations (Table 1). Analysis of comorbidities revealed that 123 (63%) were smokers, 22 (11%) alcohol dependant, 60 (31%) had mental health problems, and 35 (18%) had a history of self-harm. Only three had a current wound, these were: a venous ulcer secondary to drug use and two self-harm wounds, one of which was a deep cavity that had required hospitalization. The data also showed that over 30 (16%) had previously experienced traumatic stab wounds.

**Table 1.** Ethnicity (*N* = 87) Prisons

*Ethnicity*		
	N	*%*
Caucasian	156	83.4
Dark skinned (African or African descent)	20	10.7
Asian	5	2.7
Other	6	3.2

Risk factor analysis revealed that increasing age and increased immobility were significantly associated with increased risk of CO, while obesity and ethnicity were not (Table 2) However, these results must be viewed with caution as the sample size is small and the confidence intervals (CIs) are wide (Table 2).

**Table 2.** Risk Factors Associated with Chronic Edema (*N* = 95) Prisons

*Factors associated with the presence of chronic edema*						
	*No edema*		*Chronic edema*			
	N	*%*	N	*%*	*OR (95% CI)*	p
	175		20			
Age						
<40	50	61.49	3	15.0	1.00	
40–49	37	21.26	8	40.0	7.71 (1.94–30.61)	0.019
50+	30	17.24	9	45.0	10.70 (2.72–42.02)	
Ethnicity						
Caucasian	139	83.23	17	85.0	1.00	
Dark skinned (African or African descent)	18	10.78	2	10.0	0.91 (0.19–4.26)	0.976
Other	10	5.99	1	5.0	0.82 (0.10–6.78)	
Obesity						
Under/normal weight	127	72.57	11	55.0	1.00	
Obese/morbidly obese	48	27.43	9	45.0	2.16 (0.84–5.55)	0.108
Lower limb mobility						
Walks unaided	174	99.4	17	85.0	1.00	
Walks with aid	1	0.6	3	15.0	30.77 (3.03–312.5)	0.004

CI, confidence interval; OR, odds ratio.

## Conclusion

The study revealed that CO is not commonly encountered within a prison population with a younger age distribution. However, in older inmates with reduced mobility the risk increased. As was predicted smoking was a major factor for 123 (63%) of inmates predisposing them to the development of cardiovascular disease and cancer and 22 describing themselves as alcohol dependant. Mental health illness was a major problem affecting nearly one third (60) with 35 stating that they self-harmed and a further 30 reporting previous traumatic stab or gunshot wounds.

## Discussion and Challenges

Despite the excellent support from the prison health care services in undertaking this study, many challenges were faced and are discussed below.

- A high turnover of inmates—the prison is a short-term prison that also functions as a triage prison—inmates are referred to the prison before being transported to their final prison destination. This results in many inmates staying for one night only and limited the ability to recruit them to the study.
- Limited number of health care rooms to assess patients; therefore, two wings were selected which have an attached treatment room. Selection of two wings only may also influence sample bias. However, prisoners of all categories were housed in all the residential wings suggesting that they are a similar population. The recruitment from only two wings limited the number screened and also affects the generalizability of the data to a wider prison population.
- At the time of the study, health screens were not compulsory, which limited the number of inmates who were willing to participate in the study.
- Primary screens of inmates at arrival to the prison were not suitable for the recruitment due to many arriving under the influence of alcohol/drugs or in a very low mood and therefore unsafe for informed consent.
- Recruitment issues—the need for secondment of an existing member of staff who was familiar with the facility and prison policies for health care screening and accessing inmates for the study.
- Extension of the study duration was required to capture the study sample, which has implications for future research funding agencies.

## Case Study 2: Nursing and Residential Homes

### Background

Nursing homes in the United Kingdom are often private businesses and employ a registered nurse to oversee the care of residents as defined below:

“*Care homes for nursing care, sometimes known as nursing homes, are mainly for people who need 24-hour support, and regular care tasks carried out or supervised by a qualified nurse*.”^6^

Access to specialist advice for people with wounds and CO in nursing homes is highly variable across the United Kingdom due to many reasons, including contractual arrangements with the NHS. Residents are often the most vulnerable in the health community with complex comorbidities, including reduced mobility with many either bed or chair bound. Cognitive impairment due to problems such as dementia is a common problem.

In the United Kingdom, residential homes (also called care homes) offer accommodation for individuals who require extra help to live independently. Residents may require help with personal care, washing, or dressing. Care is provided by carers many of whom have limited training.

Residential homes are defined as:

*“A care home is a residential setting where a number of older people live, usually in single rooms, and have access to on-site care services. A home registered simply as a care home will provide personal care only—help with washing, dressing and giving medication.”*^7^

In Australia the Limprint partner organization Aged Care Housing (ACH) Group is a not for profit organization accredited by the Government to provide residential aged care. Not-for-profit and private organizations are the main providers of residential aged care services in Australia, with 60% and 30% of facilities, respectively.^8^

Residential care is defined as personal care or nursing care, or both personal care and nursing care, that:

(a) is provided to a person in a residential facility in which the person is also provided with accommodation that includes:
(i) appropriate staffing to meet the nursing and personal care needs of the person;
(ii) meals and cleaning services; and
(iii) furnishing, furniture, and equipment for the provision of that care and accommodation.^9^

In Australia the most common age at admission to an aged care facility is 85–89 years for both males and females, followed by the 80–84 age group.^10^ Increasingly those people who come into residential aged care facilities have complex comorbidities and often present for palliative services. On June 30, 2011, over three-quarters of residents (78%) were reported to have a mental illness. More than half (52%) of the 164,116 permanent residents had a diagnosis of dementia, and over two fifths of residents with dementia also had a diagnosis of a mental illness. A further 26% of residents had a diagnosis of mental illness without a diagnosis of dementia. Other common conditions were circulatory system diseases (24%), diseases of the musculoskeletal system and connective tissue (18%), and around 8% of residents had endocrine, nutritional, and metabolic disorders (such as diabetes). The average length of stay for those leaving residential care in 2010–2011 was 145.7 weeks with females on average staying around 54% longer than males (168.1 compared with 109.5 weeks).^8^

It was deemed critical as part of the LIMPRINT program to try and establish the number of people affected with CO and wounds in residential care homes to make recommendations for improved services and to inform training for staff.

### Setting

The study was undertaken in residential homes within one area in the East Midlands (United Kingdom) and two aged care facilities in Adelaide (Australia). The residential home study was part of a wider wound and CO study in the United Kingdom. The wider United Kingdom prevalence study was evaluated by the local Research and Governance department and was considered a “service evaluation.” Community nurses' who normally visit and provide care for the patients in the residential homes were responsible for obtaining informed consent from participants.

In Australia two recent physiotherapy graduates were trained to gain verbal consent and administer the core, wound, and swelling Limprint tools. They attended two metropolitan ACH Group facilities with a combined total of 252 residential beds. Both provide care to people with all levels of support needed. A verbal consent was obtained from each participant in both the U.K. and Australian studies. Participants who were unable to consent were excluded.

### Identification of participants

Specialized Care Home Community Nursing Teams undertook the screening and evaluation of all patients who were able to consent in the U.K. study. They were all fully trained in all the screening requirements. Inter-observer error was assessed by 3 practitioners (a community nurse, a tissue viability nurse [gold standard], and a researcher) on a total of 10 randomly selected patients. The presence of pitting, hardness, and Stemmer's sign was identical for all three observers. For shape distortion one practitioner was in disagreement with the other two on two occasions, giving an overall agreement of 86.7%. The free marginal Fleiss' Kappa statistic was 0.733 for shape distortion indicating a good agreement between the three raters. Based on the interobserver error study, the researchers were confident that community nurses can recognize symptoms of CO.

In Australia the physiotherapists attended a day of training to ensure consistency in their assessment. They undertook the consent and screening procedures. Results from the Australian study will be published separately.

## Results

The following results are from the U.K. nursing homes and those from the Australian residential aged care facilities. In the United Kingdom, of the total population available for inclusion in the study (*N* = 189), only 137 patients were recruited. Sixteen patients were excluded from the study for various reasons, for example, hospital admission, death not due a nursing review that week, and refusal to participate in the study with 36 patients excluded due to their inability to consent.

Seventy-two of the 137 patients screened (52%) were defined as suffering from CO, and a further 16 (23%) had a history of cellulitis. The majority of residents were Caucasian 127 (93%), and 8 (6%) were black (Table 3). Comorbidities were similar in both groups although diabetes, heart failure, neurological disorders, and peripheral arterial occlusive disease were more common in the CO group; however, this difference did not reach a standard level of statistical significance (Table 4). Wounds affected both groups (62) with a nonsignificant difference between those with CO 36 (50%) and those without 26 (40%) (*p* = 0.240). Leg ulceration was significantly likely to be associated with the presence of CO (*p* = 0.026).

**Table 3.** Ethnicity (*N* = 37) Residential Homes

*Ethnicity*		
	N	*%*
Caucasian	127	92.7
Dark skinned (African or African descent)	8	5.8
Asian	0	0
Other	1	0.7
Mixed	1	0.7

**Table 4.** Comorbidities (*N* = 37) Residential Homes

*Comorbidities*						
	*No edema*		*Chronic edema*			
	N	*%*	N	*%*	*OR (95% CI)*	p
Diabetes						
Absent	52	80.0	49	68.1	1.00	
Present	13	20.0	23	31.9	1.88 (0.86–4.11)	0.111
Heart failure/CHD						
Absent	53	81.5	54	75.0	1.00	
Present	12	18.5	18	25.0	1.47 (0.65–3.35)	0.354
Neurological disease						
Absent	47	72.3	47	65.3	1.00	
Present	18	27.7	25	34.7	1.39 (0.67–2.88)	0.375
Peripheral arterial D.						
Absent	64	98.5	69	95.8	1.00	
Present	1	1.5	3	4.2	2.78 (0.28–27.44)	0.349
Smoking						
No	64	98.5	72	100.0	1.00	0.474^a^
Yes	1	1.5	0	0		

^a^ Fisher's exact test.

CHD, chronic heart disease.

Logistic regression analysis revealed a number of potential factors associated with CO; however, these must be viewed with caution due to the small sample size, and the results are not statistically significant. CO was associated with the very elderly (over 90 years) (odds ratio [OR] 1.50, CI 0.59–3.78, *p* = 0.55), and being obese or morbidly obese (OR 5.33, CI 0.90–31.44, *p* = 0.133), (Table 5).

**Table 5.** Risk Factors for Chronic Edema (*N* = 37) Residential Homes

*Factors associated with the presence of chronic edema*						
	*No edema*		*Chronic edema*			
	N	*%*	N	*%*	*OR (95% CI)*	p
Total	65		72			
Gender						
Male	18	27.7	19	26.4	1.00	
Female	47	72.3	53	73.6	1.07 (0.50–2.27)	0.864
Age						
<80	14	21.9	13	18.3	1.00	
80–89	27	42.2	26	36.6	1.04 (0.41–2.62)	0.557
90+	23	35.9	32	45.1	1.50 (0.59–3.78)	
Ethnicity						
White	61	93.9	66	91.7	1.00	
Black	4	6.2	4	5.6	0.92 (0.22–3.86)	0.91
Other	0	0	2	2.8	—	
Obesity						
Underweight	12	19.1	9	12.5	1.00	
Normal weight	49	77.8	55	76.4	1.50 (0.58–3.85)	0.133
Obese/morbidly obese	2	3.2	8	11.1	5.33 (0.90–31.44)	
Lower limb mobility						
Bed bound	4	6.2	7	9.7	1.00	
Chair bound	24	36.9	20	27.8	0.48 (0.12–1.86)	0.549
Walks with aid	29	44.6	38	52.8	0.75 (0.20–2.80)	
Walks unaided	8	12.3	7	9.7	0.50 (0.10–2.46)	
Upper limb mobility						
Full range	23	35.9	35	50.0	1.00	
Limited	39	60.9	31	44.3	0.52 (0.26–1.06)	0.148
No function	2	3.1	4	5.7	1.31 (0.22–7.77)	

## Conclusion

The study revealed that CO is commonly encountered within residential home populations. However, the study sample is significantly skewed due to issues of recruitment bias. As was predicted comorbidities are common, and concurrent wounds affect half the patient population.

## Discussion and Challenges

This study proved one of the most challenging populations to obtain accurate information from within the LIMPRINT program. Many issues influenced the outcome and are discussed below:

- Issues of consent and capacity related to the high rates of dementia and mental health issues
- Family consent was not possible on many occasions and would require increased time and funding in future studies
- Gatekeeping by staff to exclude unwell and palliation residents
- Gatekeeping by staff who lack the understanding of those suitable to be recruited despite training
- Professional knowledge “our patients do not have chronic edema”
- Fear of scrutiny and issues of litigation (pressure ulceration)
- Capacity and impact on care staff during the study
- Balancing ethical dilemmas with establishing care needs

### Issues of consent/family consent

The issues of informed consent are of paramount importance in undertaking ethical research. Research must aim to maximize the benefits for individuals and society while minimizing the risk of harm. This involves protecting both the rights and dignity of individuals and requires that participation is voluntary and based on informed consent. This study highlighted the challenges of addressing these issues. Many patients lacked the capacity for consent, and there was a limited time to gain family consent. Frequently the families could not see the potential for their relative to be included in the study as it would not improve their care or prognosis. In addition, care staff acted as gatekeepers to the nurses undertaking the study for a number of reasons, including the pressure of time. The consequence of this is that the true burden of CO and wounds remains obscured and its potential importance underestimated.

Future studies should incorporate consent processes that are acceptable to the human research ethics committee for recruitment of people with dementia. This may involve guardian or family consent or the registered nurse. Further establishment of whether the observation and assessment of edema is usual clinical care, which is already documented in the clinical record of the partner organization, is required.

### Gatekeeping by staff/professional knowledge “our patients do not have chronic edema”

Participation of the nursing homes in this study proved challenging with four of the six nursing homes approached declining to be involved. It required agreement and cooperation with the care managers and staff. Managers did not see the importance or relevance in conducting a prevalence study in their nursing home because in their view their patients did not suffer from CO despite the researcher explaining that edema was only accurately identified through a physical assessment. As well the timing of data collection, which in some cases coincided with Christmas activities at the residential care facilities in Australia which limited flexibility to participate and a significant amount of lead up time, is required to inform staff and residents about the study requirements.

### Fear of scrutiny and issues of litigation (pressure ulceration)

Residential aged care in Australia is tightly governed with site audits. Some staff expressed concern that this was yet another audit process. U.K. managers expressed concern that the study was a method of evaluating the standards of care and were wary of this occurring. This is not surprising in a litigious public arena where issues such as pressure ulceration are often considered markers of quality of care and the development of pressure ulceration has led to litigation in recent years.

### Capacity and impact on care staff during the study

Like other nursing professionals, nursing home staff are very busy. In the United Kingdom although the research team explained that a tissue viability nurse would come in and assess all the patients and therefore the study would not impact on the workload of their staff, the managers perceived the study as an added work to their already busy staff. The employment of dedicated assessors in Australia overcame this issue.

### Balancing ethical dilemmas with establishing care needs

Researchers must find a balance between doing ethical research (excluding patients who are unable to consent or who are too sick) versus collecting data on as many patients as possible (prevalence studies). The data able to be collected during this study are not likely to be a true representative sample of all people who do and do not have CO in the residential aged care setting for all the reasons described above. Further research which targets this setting specifically and includes the suggestions discussed is needed to overcome the limitations identified. In the meantime, meaningful data are not available to support the development of effective CO services and ensure that the human rights of patients are protected. While CO remains cloaked in these complex issues the true perspective is obscured; it is possible for health care agencies and providers to ignore the urgent imperative for improved care. In some countries ethical frameworks allow the appointment of a health care proxy for those who have lost capacity for consent, which differs from the more general rules of guardianship. However, this may also be problematic as the proxy may not hold views that are representative of the patients. They may be overprotective of including patients or hold diverse views on what constitutes beneficial research.

Similarly, the research team attempted to recruit nursing homes for the U.K. study as mentioned above. To access residents in a nursing home it was necessary to obtain the relevant NHS Research Ethics and R&D approvals and the approval of the nursing home managers. Since the majority of nursing home residents suffer from long-term conditions such as dementia or learning disability, a written informed consent from a family member is frequently needed. Difficulties of obtaining approval of nursing home managers and time constraints resulted in the decision not to continue with recruitment of nursing homes by the research team. The research team however hopes to access this under-researched patient population in the future.

Despite the desire to undertake a study within mental health inpatient services, this was not possible. The main reasons for this were not only issues of capacity for consent by patients but also the safety for staff who were not mental health trained. Mental health staff in this project felt that they did not see CO in their patients. They did acknowledge that this was not routinely assessed. Anecdotal information from Lymphedema services in the United Kingdom has shown that patients with mental health issues do present with CO that may be exacerbated by neuroleptic medications. A similar picture was seen when attempts were made to access people receiving social rather than nursing care in their own homes.

### General reflections

The undertaking of LIMPRINT internationally has raised many practical and complex ethical dilemmas. Despite all sites using the same study protocol, it was perceived in some countries as noninterventional and part of a service evaluation, while in others a full ethical approval was required. Data collection methods and the storage of data on servers held outside of Europe prevented some sites from being able to use this facility. The reality of undertaking international research requires a complex knowledge of these issues to maximize successful outcome.
